# AIF1L as a Ferroptosis-Linked Biomarker in Microsatellite States–Driven Colorectal Cancer: Functional and Diagnostic Insights From Multiomics Analysis

**DOI:** 10.1155/humu/6663166

**Published:** 2025-10-10

**Authors:** Yuanyuan Qin, Hongli Zhou, Lingyan Zhu, Wenting Li, Zequn Jiang, Li Li, Mianhua Wu

**Affiliations:** ^1^The First Clinical Medical College, Nanjing University of Chinese Medicine, Nanjing, Jiangsu, China; ^2^Jiangsu Collaborative Innovation Center of Traditional Chinese Medicine Prevention and Treatment of Tumor, Nanjing University of Chinese Medicine, Nanjing, Jiangsu, China; ^3^School of Medicine, Nanjing University of Chinese Medicine, Nanjing, Jiangsu, China

**Keywords:** AIF1, colorectal cancer, microsatellite instability, prognosis, tumor microenvironment

## Abstract

**Background:**

Microsatellite instability (MSI) serves as a crucial biomarker for immune checkpoint blockade therapy in colorectal cancer (CRC). However, only around 40% of MSI CRC patients benefit from ICB. Investigating the mechanisms underlying MSI CRC, particularly its association with cell death and the immune microenvironment, can provide insights to improve immunotherapy efficacy.

**Methods:**

Transcriptomic and clinical data of MSI CRC patients were collected from TCGA and GEO databases. Differential expression analysis, weighted gene coexpression network analysis, and Cox regression models were employed to identify five cell death–related prognostic genes (POU4F1, AIF1L, SLC18A1, INSL4, and HOXC6). Single-cell analysis, immune infiltration analysis, and in vitro and in vivo experiments were conducted to validate their roles in MSI CRC.

**Results:**

The five-gene risk model effectively stratified high- and low-risk groups and predicted survival differences (AUC > 0.6). AIF1L exhibited elevated expression in MSI-H groups and demonstrated a significant correlation with ferroptosis and immune cell infiltration. In vitro experiments showed that AIF1L boosted cell proliferation and migration via modulating ferroptosis, showing correspondence with in vivo experiments. Moreover, enrichment analysis revealed that AIF1L participated in immune-related signaling pathways, potentially impacting the tumor microenvironment and patient prognosis.

**Conclusion:**

AIF1L may regulate MSI CRC progression by promoting ferroptosis, serving as a prospective biomarker for prognosis and a therapeutic target for personalized therapy. This study uncovers new mechanisms in MSI CRC and provides a foundation for optimizing immunotherapy, though further investigation into its specific roles is needed.

## 1. Introduction

Colorectal cancer (CRC), which encompasses colon and rectal cancers, is the second leading cause of cancer-related mortality and the third most prevalent cancer worldwide [1]. It is reported that in 2022, there were approximately 2 million new cases of CRC and more than 900,000 deaths from CRC worldwide [1]. The incidence and mortality rates of CRC generally rise with advancing age, and the occurrence of early-onset CRC has surged significantly [2]. Currently, surgery, chemotherapy, radiotherapy, and targeted therapies are the main treatments for CRC [3]. Despite recent advancements in surgical techniques and adjuvant therapies, the survival rate for patients with CRC remains low, and the likelihood of postoperative recurrence and metastasis is elevated [4]. Consequently, CRC remains a major public health challenge.

CRC is a heterogeneous disease driven by instability of the mutant genome [5]. It is currently believed that CRC is developed primarily through three mechanisms: chromosomal instability, microsatellite instability (MSI), and CpG island methylator phenotype [6]. Clinically, MSI CRC is predominantly located in the right colon, with a lower clinical grade and improved prognosis, characterized by mucinous characteristics, extensive lymphocyte infiltration, and Crohn-like symptoms [5]. Due to its elevated mutational burden and neoantigen generation, MSI CRC demonstrates significant efficacy against immune checkpoint inhibitors (ICIs) [7]. For example, the efficacy of ICIs is significantly enhanced when ferroptosis is induced, depending on the activation of antitumor immunity [8]. Nonetheless, MSI CRC can achieve drug resistance through immunosuppressive methods within the related-signaling pathway in the tumor microenvironment [9]. Consequently, enhancing immunotherapy efficacy in the CRC patients is essential.

**Figure 1:**
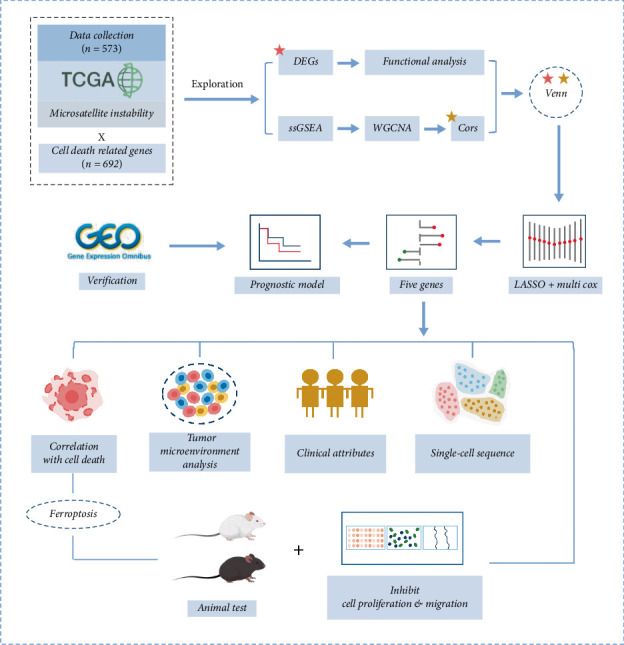
The overall workflow of this study.

The molecular expression profiles of CRC have been extensively investigated owing to advancements in sequencing technologies, facilitating the application of molecular biomarkers as prognostic indicators, diagnostic tools, and therapeutic targets for CRC patients. In this study, expression matrices of MSI CRC were obtained from TCGA and GEO databases, and an integrative approach combining bioinformatics analyses with in vitro experiments was employed to identify reliable molecular targets for MSI CRC monitoring. Furthermore, single-cell transcriptomics and CNV inference were incorporated to provide additional mechanistic insights into tumor biology. The findings of this work are expected to provide novel perspectives on disease prognosis, anticancer therapy, and immunotherapy sensitization in CRC. An overview diagram of the study is presented in Figure 1.

## 2. Materials and Methods

### 2.1. Data Collection

Transcriptomic data (mRNA sequence [the mRNA-seq]), clinical information, and associated MSI information were collected from TCGA (https://portal.gdc.cancer.gov/repository). In TCGA-COAD and TCGA-READ cohorts, there were 573 samples with MSI information, and we defined the MSI default threshold > 0.4 as MSI-H (*n* = 92) and the MSI default threshold < 0.4 as microsatellite stable (MSS, including MSI-L, *n* = 481). Moreover, GSE29621, extracted from the GEO database (https://ww.ncbinlm.nih.gov/), included 65 CRC samples with survival information [10]. GSE166555, a single-cell dataset extracted from the GEO database, contained 13 CRC samples alongside 12 controls [11].

Besides, we collected 692 cell death–related genes, including anoikis-related genes from prior literature [12], apoptosis-related genes from the published literature [13], autophagy-related genes cataloged in the human autophagy database (HADb, http://www.autophagy.lu/), cuproptosis-related genes from FerrDb (http://www.zhounan.org/ferrdb/current/), disulfidptosis-related genes from FerrDb, entosis-related genes from the GeneCards Database (https://www.genecards.org/), ferroptosis-related genes from FerrDb, necroptosis-related genes from the published literature [14], oncosis-related genes from the GeneCards Database, paraptosis-related genes from the GeneCards Database, pyroptosis-related genes from the existing literature [15], and PANoptosis-related genes gathered from the published literature [16].

### 2.2. Differentially Expressed Analysis

Differential analysis between MSI-H and MSS groups was conducted utilizing the “DESeq2” R package (Version 3.44.3), with |log_2_foldchange (FC)| > 1 and adjusted *p* value < 0.05 [17]. Moreover, differentially expressed genes (DEGs) were displayed through the volcano plot and heatmap.

### 2.3. Functional Analysis

Gene Ontology (GO) and Kyoto Encyclopedia of Genes and Genomes (KEGG) analyses were conducted to explore the biological processes and pathways linked to the DEGs via R package “clusterProfiler” (Version 3.16.0) [18]. *p* < 0.05 was deemed significantly enriched.

### 2.4. Weighted Gene Coexpression Network Analysis (WGCNA)

Cell death scores for samples in TCGA-COAD and TCGA-READ cohorts were calculated via single-sample gene set enrichment analysis (ssGSEA) [19]. WGCNA, an algorithm capable of identifying coexpression relationships between genes and discovering genes associated with specific phenotypes through modular analysis [20], was then applied to identify genes correlated with MSI and cell death. We set the power value as 7 to complete the process, and modules selected for further analysis needed to satisfy the following requirements: (a) it is not a gray module, (b) the *p* value was lower than 0.05, and (c) the absolute value of the correlation coefficient is greater than 0.3. These genes in selected modules were marked as microsatellite instability and cell death–related genes (MSI-CDRGs).

### 2.5. Construction and Validation of a Risk Model

In TCGA-COAD and TCGA-READ cohorts, 474 samples with survival data were chosen and randomly allocated into TCGA training and TCGA validation cohorts (7:3 ratio). Moreover, GSE29621 was the external validation cohort. Firstly, DEGs intersected MSI-CDRGs to obtain intersection genes. In TCGA training cohort, the genes related to prognosis in intersection genes were selected through combining univariate Cox regression analysis (hazard ratio [HR] ≠ 1 and *p* < 0.05), least absolute shrinkage and selection operator (LASSO) Cox regression, and multivariate COX (HR ≠ 1 and *p* < 0.05). The risk scores were computed following the formula: risk score = 0.0016∗POU4F1 + 0.047∗AIF1L + 0.014∗SLC18A1 + 0.026∗INSL4 + 0.039∗HOXC6 (gene expression). Patients were then classified into high- and low-risk groups by the median value of risk scores. Overall survival (OS) differences were measured via Kaplan–Meier (K-M) curves, and the predictive capability of the risk model was evaluated by receiver operating characteristic (ROC) curves. Finally, TCGA validation cohort and GSE29621 were utilized for verification to enhance the model's credibility.

### 2.6. Correlation Between Cell Death and Prognostic Genes

To investigate correlations between different types of cell death and risk score, each cell death type score was calculated via ssGSEA based on each cell death type related genes in TCGA training cohort. Then, differences between the two groups were analyzed, and correlations between prognostic genes and different types of cell death that showed significant differences between the two groups were measured via the Pearson algorithm. Types of cell death included anoikis, apoptosis, cuproptosis, entosis, ferroptosis, necroptosis, autophagy, oncosis, paraptosis, disulfidptosis, pyroptosis, and PANoptosis.

### 2.7. Gene Set Enrichment Analysis (GSEA)

The “clusterProfiler” R package was employed to perform GSEA to investigate the cell death–related prognostic genes enriched biological processes and signaling pathways. Reference sets “c2.cp.kegg.v7.5.1.entrez.gmt” and “c5.all.v7.5.1.entrez.gmt” were obtained from the Msigdb database. The thresholds were |NES| > 1 and adjusted *p* value < 0.5.

### 2.8. Immune Landscape Analysis

The immune landscape in two groups was evaluated with the ESTIMATE algorithm (Version 1.0.13) and ssGSEA. ssGSEA helps identify active biological processes or pathways in different samples. The immune score, stromal score, ESTIMATE score, and tumor purity were evaluated through the ESTIMATE algorithm according to the ratio of immune cells to stromal cells. The Wilcoxon test was utilized to ascertain the distinctions between the two groups. Additionally, the Spearman algorithm was employed to quantify correlations between immune cells and prognostic genes. Moreover, the TIDE score was utilized to predict the effectiveness of ICI therapy in patients categorized into two risk groups.

### 2.9. Correlations Between Risk Model and Clinical Characteristics

To measure whether there were differences in clinical characteristics (including gender, age, stage, pathological-T, pathological-M, and pathological-N) between the two groups, the chi-square test was utilized. Univariate and multivariate Cox were then employed to ascertain whether the risk score served as an independent prognostic indicator for OS in TCGA training cohort. A nomogram is a statistical tool that combines multiple prognostic factors to provide individualized survival predictions. Subsequently, based on independent prognostic factors, a nomogram was invented to forecast the survival of MSI CRC patients at 1, 3, and 5 years. And the predictive effect of the nomogram was validated through plotting calibration curves and decision curve analysis (DCA).

### 2.10. Single-Cell Analysis

The “Seurat” R package (Version 4.4.0) was used to filter valid cells based on criteria including a minimum of three cells and 200 features, with additional filtering for library size, gene count, and mitochondrial content. After normalization, the Top 2000 highly variable genes were extracted by the vst method of the FindVariableFeatures function. Principal component analysis (PCA) was applied, followed by unsupervised clustering using FindNeighbors and FindClusters. The marker genes for each cell type were compared with those in the CellMarker database to ascertain the cell type of the cell clusters [21]. Thereafter, the percentage of each cell type in CRC and control samples was evaluated. The expression of prognostic genes was examined in each cell type of CRC and normal groups. Additionally, CNV inference was performed to assess genomic alterations in epithelial cell subtypes (inferCNV R package, Version 1.22.0). Normal epithelial cells were set as the reference, and CNV profiles were visualized on UMAP plots to distinguish diploid and aneuploid states between tumor and normal groups.

### 2.11. Pseudotime Analysis and Cell–Cell Communication Analysis

The Monocle2 (Version 2.14.0) was employed to carry out pseudotime analysis, a method used to model dynamic changes in cell states, based on the following three steps: gene screening, dimensionality reduction, and cell sorting. The CellChat V2 [22] was employed to perform cell–cell communication analysis.

### 2.12. Cell Culture and Transfection

The human MSI-H line HCT116, MSS CRC line HT29, mouse MSI-H line MC38, and MSS CRC line CT26 were purchased from Wuhan Shangen Biotechnology Co. Ltd. and stored in RPMI 1640 (KGL1501-500; Keygen BioTECH) with 10% FBS (Moregate bitech, Australia) under 37°C and 5% CO_2_. The small interfering RNA were synthesized by Corues (Nanjing, Jiangsu, China). The siRNA sequences were enumerated below: Si-AIF1L: 5⁣′-AGCUGGUCAUGAUGUUUGA-3⁣′; negative control: 5⁣′-UUCUCCGACAGUGUCACGU-3⁣′.

### 2.13. Quantitative Real-Time Polymerase Chain Reaction (RT-qPCR)

The total RNAs were extracted with TRIzol (Invitrogen, CA, United States), and the concentration was detected by NanoDrop. Next, the RNA was converted into cDNA with the corresponding kit (ThermoFisher, K1622). After that, SYBR Green Master Mix (Thermo Fisher) was employed to perform RT-qPCR according to the cycler protocol. Supporting Information 3: Table S1 listed the primer sequences.

### 2.14. Cell Proliferation Assay

For cell counting kit-8 assay, 5 × 10^3^ cells (MC38 and CT26 cell lines) were placed into 96-well plates, followed by 10 *μ*L CCK-8 reagent (K1018, APExBIO) in each well. The optical density value at 450 nm was measured by a multifunctional microplate reader (Spark10M, Tecan, Switzerland).

To further understand the cell growth, the EdU cell proliferation assay was also carried out in accordance with the instructions of the EdU Elab Fluor 488 Imaging Assay Kit (Elabscience Biotechnology Co. Ltd.). The results were evaluated utilizing an inverted fluorescence microscope (ECLIPSE Ts2, Nikon, Japan).

### 2.15. Wound Healing Cell Migration Assay

MC38 and CT26 cells were seeded in six-well plates containing scratch inserts (Coolrun, CR211-2) and cultured in standard medium until the cells just attached to the wall. Subsequently, the inserts were removed with tweezers, and serum-free medium was introduced. Images were captured at 0 and 48 h by utilizing a microscope and measuring scratch width by applying the ImageJ 1.51 software.

### 2.16. Construction of Animal Model and Experiment Design

Male BALB/c and C57BL/6J mice (18–22 g, nine mice per group) were obtained from Jiangsu Qinglongshan Biotechnology Co. Ltd. The study adhered to the criteria established in the Animal Research: Reporting of In Vivo Experiments (ARRIVE), and the experimental protocol was authorized by the Animal Ethics Committee of Nanjing University of Chinese Medicine (Approval Number: 202407A061). Subcutaneous tumors were first established by injecting 0.1 mL of MC38 or CT26 cells (1 × 10^7^/mL) into the right armpit of one mouse per group. After 15 days, the tumors were harvested and cut into 1 mm^3^ fragments. For heterotopic implantation, mice were anesthetized with isoflurane, and a 1-mm^3^ tumor fragment was surgically implanted onto the scraped surface of the cecal serosa. The tumor was secured using medical adhesive, and the abdominal cavity was closed. After 10 days, tumors were collected for downstream analysis. A schematic overview of the animal procedure is shown in Supporting Information 1: Figure S1.

### 2.17. Western Blotting (WB)

The proteins were separated from cells and tumor tissue, applied to 20 *μ*g per sample on a 10% PAGE gel, and subsequently transferred onto 0.22 *μ*m PVDF membranes. Membranes were blocked for 1.5 h and incubated with GPX4 (1:1000; Abcam, ab197345), FACL4 (1:5000; Abcam, ab155282), and *β*-actin (1:2000; Abcam, ab125066) antibody at 4°C overnight. Next, the membranes were exposed to rabbit secondary antibody for 1 h. The protein was visualized using ChemiDoc XPS, and ImageJ 1.51 software was used for quantitative analysis.

### 2.18. Immunohistochemistry (IHC)

Tumor specimens were preserved in 4% paraformaldehyde and inserted into paraffin. After being sectioned into 5 *μ*m slices, the specimens were deparaffinized with xylene and rehydrated via a graded succession of alcohols. After treating with EDTA (Beyotime) for 15 min, 3% H_2_O_2_ for 20 min, and blocking with QuickBlock Blocking Buffer (Beyotime) for 5 min, the sections were incubated overnight at 4°C with AIF1L antibody (Solarbio, K109926P, 1:100), GPX4 antibody (Abcam, ab125066, 1:100), and FACL4 antibody (Abcam, ab155282, 1:200). Washing with PBS for three times, the sections were incubated for 30 min with ready-to-use HRP-conjugated anti-rabbit secondary antibody (Proteintech, PR30011). Then, the sections were stained with 3,3⁣′-diaminobenzidine tetrahydrochloride and counterstained with hematoxylin. Images were collected under a microscope and analyzed quantitatively using ImageJ software.

### 2.19. Malondialdehyde (MDA) and Glutathione (GSH) Assays

Approximately 1 × 10^6^ cell precipitates were collected, followed by the addition of 0.5 mL precooled PBS and grinding for 5 min on ice. Additionally, approximately 50 mg of tumor tissue was placed in a homogenizer containing nine times the volume of precooled normal saline. The homogenates were centrifuged for 10 min at a speed of 3000 rpm·min^−1^ under low temperature. An appropriate volume of supernatant was utilized for tissue protein measurement by the BCA technique. MDA and GSH content were determined according to Nanjing Jiancheng Bioengineering Institute Co. Ltd. (A003-1 and A006-2).

### 2.20. Statistical Analysis

R software (Version 4.2.1) and GraphPad Prism 8.0 were utilized to perform statistical analysis and visualization. Differences between groups were measured via the chi-square test, two-tailed paired *t*-test, Mannheimer's *U*-tests, and Wilcoxon's test. *p* < 0.05 indicated a significant difference.

## 3. Results

### 3.1. Identification of DEGs Between MSI-H and MSS

**Figure 2:**
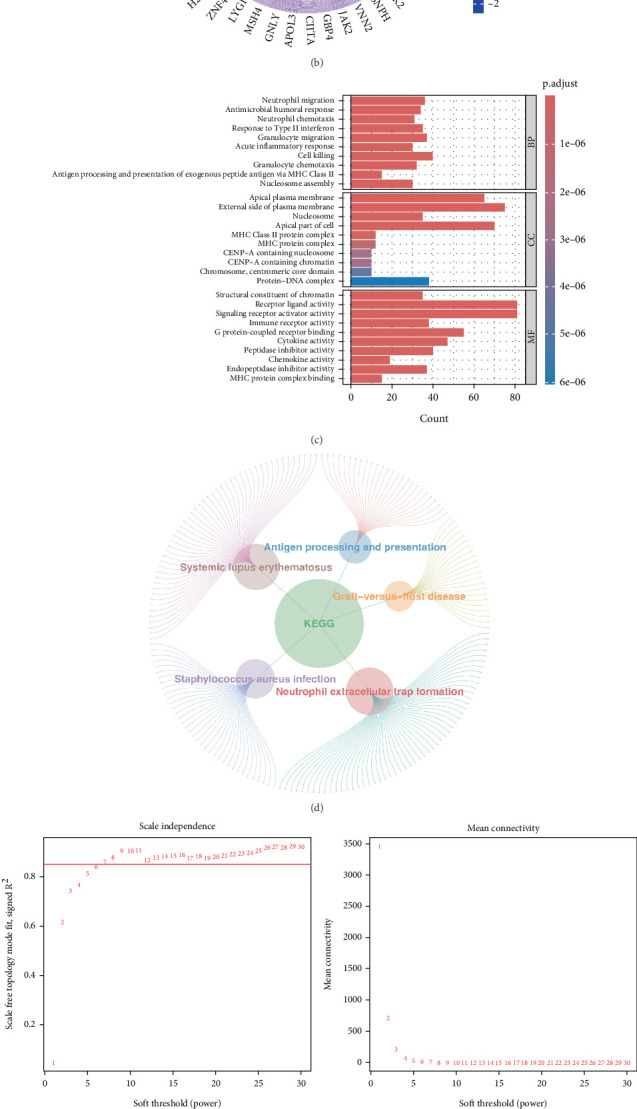
Identification of intersection genes between DEGs between MSI-H and MSS groups and MSI-CDRGs. (a) Volcano plot illustrating DEGs between MSI-H and MSS groups. (b) Heatmap showing the Top 20 upregulated and downregulated genes in DEGs. (c) GO enrichment analysis of DEGs. (d) The Top 5 functional enrichments in KEGG analysis of DEGs. (e, f) Identification of MSI-CDRGs through WGCNA. (e) Scale independence and average connectivity of various soft-thresholding powers (*β*) ranging from 1 to 30. (f) Heatmap illustrating the link between module eigengenes (MEs) and MSI-H, along with cell death. (g) Venn diagram showing the overlap of genes between DEGs and MSI-CDRGs.

To investigate the molecular variations between MSI-H and MSS, differentially expressed analysis was conducted utilizing TCGA database. In total, 1432 DEGs between MSI-H and MSS were identified, including 775 upregulated and 657 downregulated genes (Figure 2a). The Top 20 upregulated and downregulated genes were exhibited via the heatmap (Figure 2b). Subsequently, we performed functional analysis to determine their possible biological functions. Results showed that 752 GO items and 42 KEGG pathways were significantly enriched (Supporting Information 4: Table S2 and Supporting Information 5: Table S3). The Top 10 GO BP, GO CC, and GO MF items are displayed in Figure 2c, respectively. For instance, DEGs were involved in “cell killing,” “apical plasma membrane,” “structural constituent of chromatin,” and so on. As shown in Figure 2d, DEGs were involved in “*Staphylococcus aureus* infection,” “systemic lupus erythematosus,” “neutrophil extracellular trap formation,” and so on. Notably, the biological functions associated with cell death were significantly enriched, such as “cell killing” and “neutrophil extracellular trap formation,” “cell fate commitment,” “cell fate specification,” and “positive regulation of T cell apoptotic process” (Supporting Information 3: Table S1 and Supporting Information 4: Table S2). Different types of cell death patterns all have profound effects on cancer development and progression [21]. Therefore, we speculated that the expression imbalance of genes in the MSI-H group may be related to cell death.

### 3.2. Identified Intersecting Genes Between DEGs and MSI-CDRGs

To investigate the connection between cell death and MSI CRC, we performed WGCNA to identify genes that were associated with MSI and cell death. Results of WGCNA indicated that the black module (cell death score: cor = 0.78, *p* = 1e − 119; MSI-H: cor = 0.35, *p* = 5e − 18; MSS: cor = −0.35, *p* = 5e − 18), salmon module (cell death score: cor = 0.6, *p* = 6e − 56; MSI-H: cor = 0.34, *p* = 2e − 17; MSS: cor = −0.34, *p* = 2e − 17), and pink module (cell death score: cor = −0.32, *p* = 4e − 15; MSI-H: cor = −0.61, *p* = 9e − 59; MSS: cor = 0.61, *p* = 9e − 59) were selected as key modules, and 2588 MSI-CDRGs were obtained (Figure 2e,f). After that, 744 intersecting genes were obtained through the intersection between DEGs and MSI-CDRGs (Figure 2g).

### 3.3. Construction and Validation of the Risk Model

**Figure 3:**
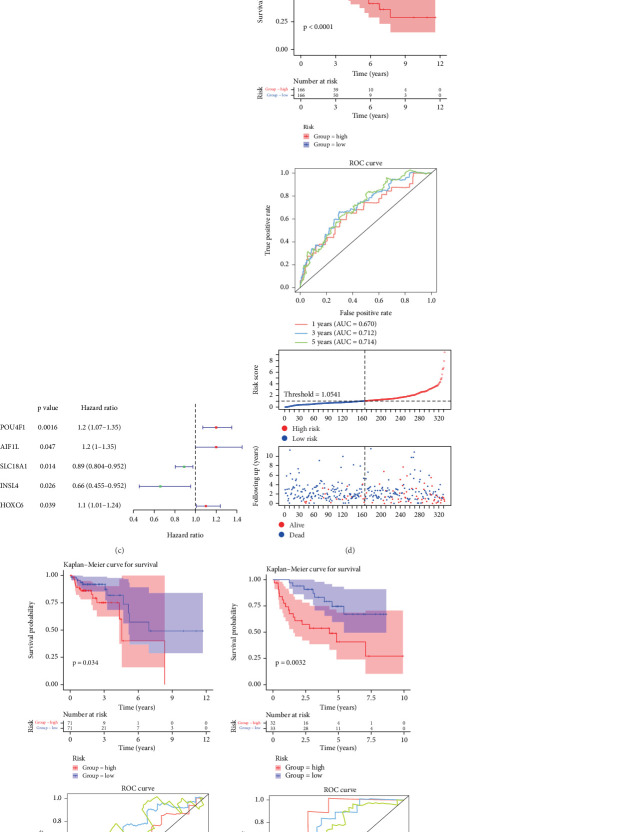
Conduction and validation of cell death–related prognostic signature for MSI CRC patients. (a) Forest plot illustrating the prognostic genes of MSI CRC patients through univariate Cox regression. (b) LASSO algorithm for selecting genes related to prognosis of MSI CRC patients. (c) Forest plot showing the genes related to prognosis of MSI CRC patients through multivariate COX regression. Development and validation of cell death prognostic risk signatures in MSI CRC (d) TCGA training cohort, (e) TCGA validation cohort, and (f) GEO validation cohort from top to bottom: K-M survival analysis of high- and low-risk groups, plots of the AUC for time-dependent ROC performance, risk score between high- and low-risk groups, and survival status of patients in high- and low-risk group.

Based on 744 intersecting genes, a total of 10 genes linked to the prognosis of CRC samples were found via univariate Cox regression analysis (Figure 3a). LASSO and multivariate Cox regression analyses [23] further selected POU4F1, AIF1L, SLC18A1, HOXC6, and INSL4 for constructing a risk model (Figure 3b,c). TCGA training cohort was stratified into high- and low-risk groups via the median risk score, with survival analysis indicating considerable disparities between the two groups (*p* < 0.0001, Figure 3d). After that, the areas under the time-dependent ROC curves (AUCs) indicated their robust prognostic efficacy of MSI CRC, with AUC values surpassing 0.6 at 1-, 3-, and 5-year intervals (Figure 3). Validation conducted on TCGA and GSE29621 cohorts demonstrated consistent results (Figure 3e,f). The findings suggest that the five-gene signature provides effective prognostic insights for MSI CRC. Therefore, these five genes were marked as prognostic genes for MSI CRC.

### 3.4. Correlation Analysis Between Cell Death and Risk Score

**Figure 4:**
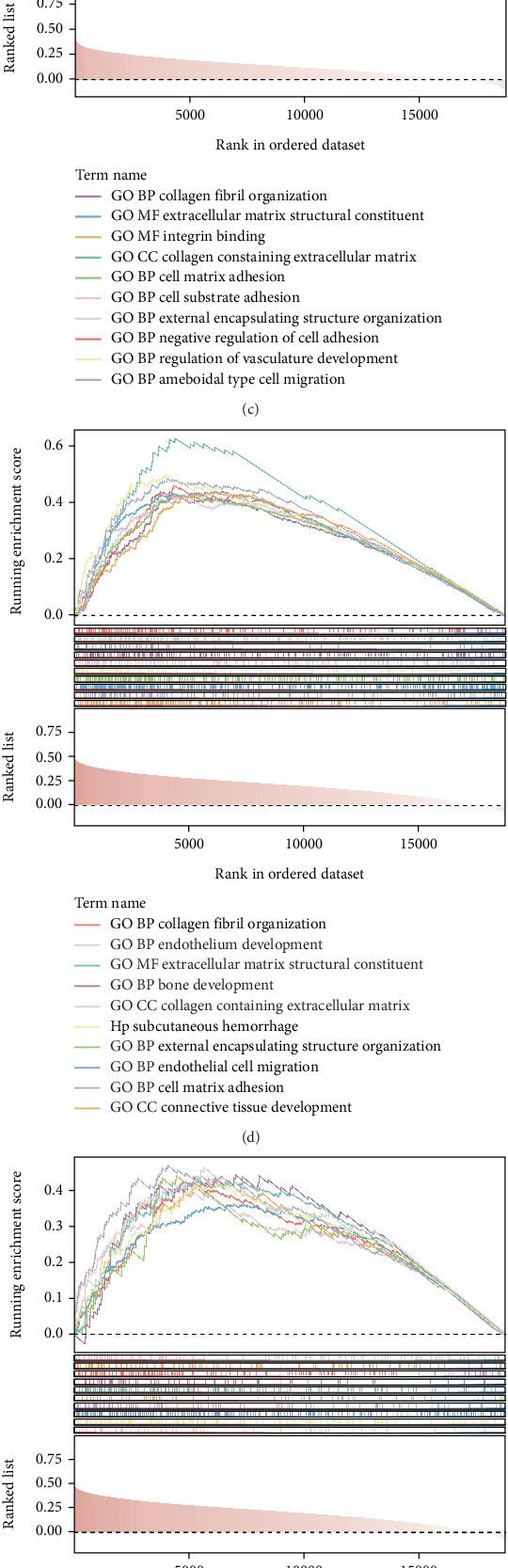
Differentially analysis in cell death score between the two risk groups and GSEA of prognostic genes. (a) Box plots manifesting the difference in cell death score between the two risk groups. (b) Heatmap illustrating the correlation between ferroptosis and prognostic genes. GO enrichment analysis of (c) AIF1L and (d) HOXC6. KEGG enrichment analysis of (e) AIF1L and (f) HOXC6. ns, not significant. ⁣^∗^*p* < 0.05, ⁣^∗∗^*p* < 0.01, ⁣^∗∗∗^*p* < 0.001, and ⁣^∗∗∗∗^*p* < 0.0001.

Through further investigating the connection between cell death and risk score, we noticed that anoikis score, autophagy score, disulfidptosis score, and ferroptosis score between the two risk groups were markedly different, and the difference of ferroptosis score between the two risk groups was most significant (Figure 4a). After that, we measured the correlation between ferroptosis and prognostic genes. Results indicated that AIF1L and HOXC6 were both significantly positively associated with ferroptosis (*p* < 0.01, |cor| > 0.3) (Figure 4b). The above results indicated the the dysfunctional expression of AIF1L and HOXC6 in MSI CRC may be associated with ferroptosis.

### 3.5. Enrichment Analysis of Prognostic Genes

The above correlation analyses underscored the importance of ferroptosis with the regulation of two genes (AIF1L and HOXC6) concerning the prognosis of MSI CRC. GSEA based on GO BP and KEGG was conducted to further clarify the prospective biological roles (Figures 4c, 4d, 4e, and 4f and Supporting Information 2: Figure S2). The GO BP results indicated that AIF1L was involved in cell-related items (e.g., “negative regulation of anoikis” and “macroautophagy”) and ferroptosis-related pathways (e.g., “regulation of cation channel activity”) (Figure 4c). Moreover, AIF1L and HOXC6 were positively associated with processes like “collagen fibril organization,” “cell-matrix adhesion,” and “extracellular matrix structural constituent” (Figure 4c,d). The KEGG enrichment analysis showed that AIF1L and HOXC6 were involved in “pathways in cancer” (Figure 4e,f). AIF1L and HOXC6 were also enriched in immune-related pathways, such as “chemokine signaling,” “ECM receptor interaction,” and “T cell activation” (Figure 4e,f). These results illustrate that AIF1L and HOXC6 have essential roles in cell death and tumor regulation. Activation or inhibition of immunological pathways may directly result in variations in antitumor immune responses, perhaps serving as a significant factor for the prognostic disparities across MSI patients.

### 3.6. Analysis of Tumor Microenvironment

**Figure 5:**
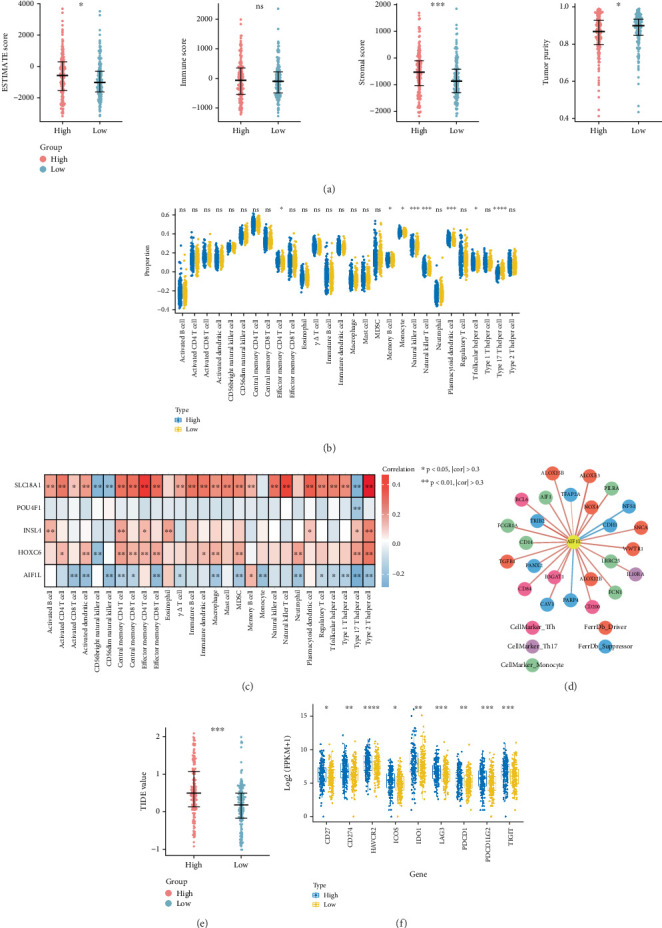
The risk score reshapes the immune cell infiltration landscape. (a) Box plots showing the ESTIMATE score, immune score, stromal score, and tumor purity between the two risk groups. (b) Box plots indicating the evaluation of immune cells between the two risk groups. (c) Heatmap illustrating the correlation between immune cells and prognostic genes. (d) Correlation network diagram showing the significant correlations between AIF1L and genes associated with ferroptosis and immune cell markers. (e) Box plot illustrating the difference in TIDE score between the two risk groups. (f) Expression levels of immune checkpoint genes in high- and low-risk groups. ns, not significant. ⁣^∗^*p* < 0.05, ⁣^∗∗^*p* < 0.01, ⁣^∗∗∗^*p* < 0.001, and ⁣^∗∗∗∗^*p* < 0.0001.

The tumor microenvironment, encompassing stromal and immune cells, significantly influences processes such as tumor progression, metastasis, recurrence, and resistance to treatment. Therefore, correlations among risk score, stromal score, ESTIMATE score, immune score, and tumor purity were systematically analyzed, revealing significant differences between the two risk groups (Figure 5a). The activity of immune cells in each sample in TCGA training cohort was assessed (Figure 5b). Effector memory CD8 T cells, memory B cells, monocytes, NK cells, natural killer B cells, plasmacytoid dendritic cells, T follicular helper cells, and Type 17 T helper cells showed statistically significant variations among the high-risk and low-risk cohorts (Figure 5b). The correlative analysis between prognostic genes and immune cells suggested that AIF1L was markedly negatively relevant to most immune cells, while SLC18A1 was significantly correlative with most immune cells (Figure 5c). We identified immune cell types (monocyte, memory B, Tfh, and Th17) significantly associated with AIF1L and their corresponding marker genes (Supporting Information 6: Table S4) and selected 14 related ferroptosis genes from the FerrDb database (Supporting Information 7: Table S5, Supporting Information 8: Table S6, and Supporting Information 9: Table S7). The correlation network showed that AIF1L was significantly positively linked to immune cell genes and ferroptosis driver genes and negatively correlated with suppressor genes (Figure 5d). Additionally, TIDE scores were calculated to assess the ICI response in the two risk groups. The findings demonstrated that the low-risk group had a significantly lower TIDE score, suggesting they are more likely to derive greater benefit from ICI therapy (Figure 5e). Interestingly, while the TIDE score was paradoxically lower in the high-risk group, immune checkpoint genes such as PD-1, PD-L1, and CTLA-4 were significantly elevated in the low-risk group (Figure 5f). This discrepancy may reflect the complex interplay between immune activation and immune evasion mechanisms captured by the TIDE algorithm.

### 3.7. Development and Calibration of the Nomogram Integrating Clinical Information and Risk Score

**Figure 6:**
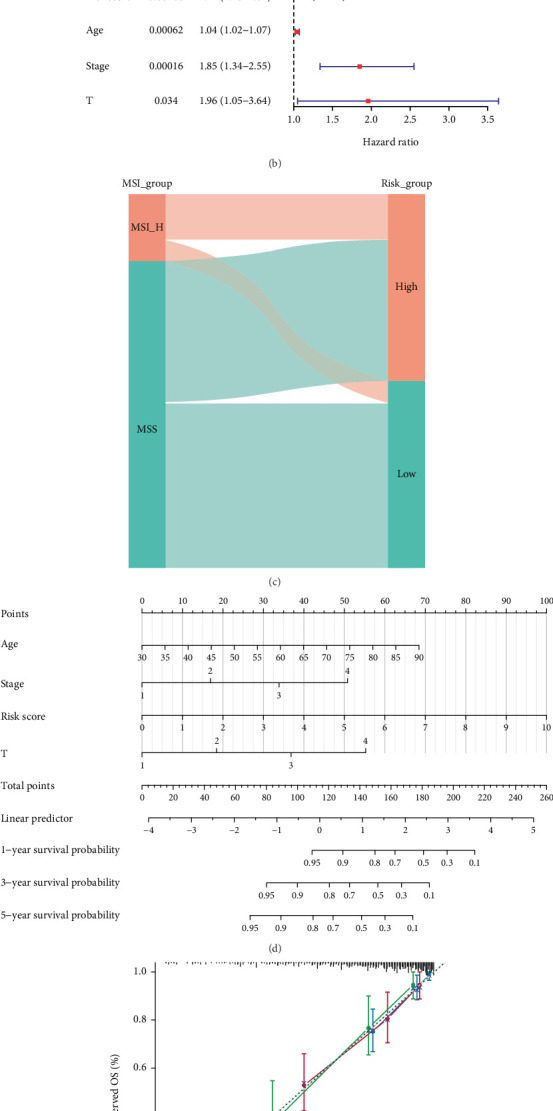
Correlation between risk score and clinical characteristics. (a) Heatmap depicting the relationship between risk score and clinical characteristics. (b) Forest plots manifesting the independent prognostic factors for prognosis of MSI CRC patients. (c) The distribution of microsatellite status across high- and low-risk groups. (d) The nomogram to predict the 1-, 3-, and 5-year OS rate of MSI CRC patients. (e) The calibration curve for assessing the precision of the nomogram model. The gray dashed diagonal line signifies the optimal nomogram. (f) DCA of the nomogram for 5 years.

To examine the correlation between risk score and clinical attributes, we performed the chi-square test and found significant differences in T, N, and stage among two risk groups (Figure 6a). Four independent prognostic factors were identified, namely, stage (*p* = 0.00016), risk score (*p* = 5.6e − 08), age (*p* = 0.00062), and pathologic-T (*p* = 0.034) (Figure 6b). As shown in Figure 6c, most MSI-H cases were classified into the high-risk group, whereas the majority of MSS cases were assigned to the low-risk group, highlighting the association between MSI status and the model-derived risk stratification. Subsequently, we further examined whether the risk model exhibited comparable or superior predictive validity relative to other independent prognostic factors based on the clinically significant prognostic qualities that varied across the two risk groups. Therefore, the nomogram was conducted to predict OS of patients with four independent prognostic factors (Figure 6d).  Figure 6e illustrated that the anticipated survival rate corresponded closely with the actual survival rate. The DCA demonstrated that the nomogram's net benefit was best (Figure 6f). The preceding results indicated that the risk score may function as an independent predictive factor or be combined with current clinical characteristics.

### 3.8. There Were 32 Cell Clusters Identified, Which Were Annotated to 11 Cell Types

**Figure 7:**
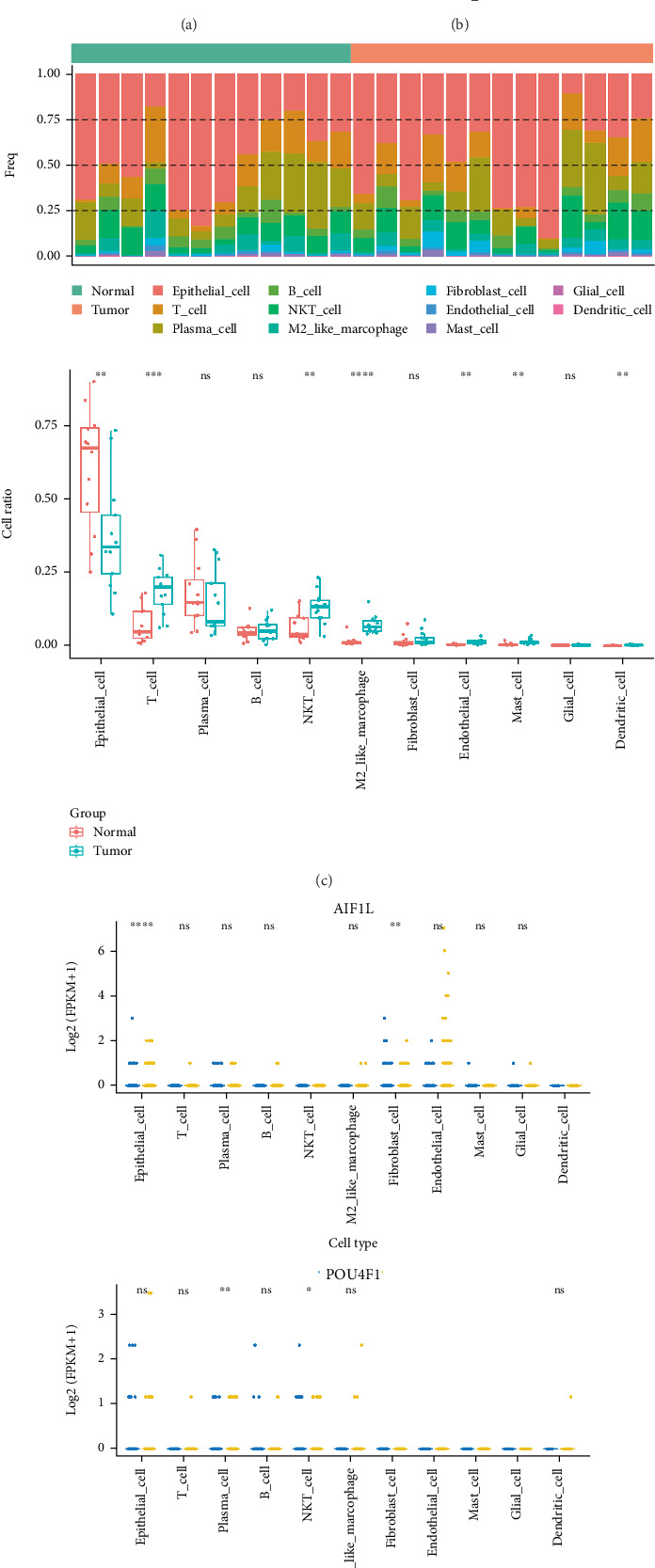
Single-cell sequence analysis. (a) t-SNE projections of single cells from CRC and normal samples, displaying 32 clusters with labels. Each point corresponds to a cluster and is colored according to the cell cluster. (b) t-SNE projections of single cells from CRC and normal samples, displaying 11 cell types with labels. Each point corresponds to a cell and is colored according to the cell. (c) Cell percentage of each cell type in each sample. From top to bottom: a bar chart; a box plot. (d) Box plots depicting the expression of prognostic genes in each cell. From top to bottom: AIF1L; POU4F1; SLC18A1, ns, not significant. ⁣^∗^*p* < 0.05, ⁣^∗∗^*p* < 0.01, ⁣^∗∗∗^*p* < 0.001, and ⁣^∗∗∗∗^*p* < 0.0001.

Single-cell analysis was utilized to investigate the expression of prognostic genes among various cell types. After filtering, the remaining cells per sample varied between 69,494 and 62,137. Following data standardization, we sought out 2000 genes with the highest variance per sample. The expression levels of these 2000 genes were dimensionally reduced to PC1–30 by PCA. A sum of 32 cell clusters was determined by unsupervised clustering analysis and were finally annotated to obtain 11 cell types, namely, epithelial cell, B cell, T cell, plasma cell, NKT cell, M2-like macrophage, fibroblast cell, endothelial cell, mast cell, glial cell, and dendritic cell (Figure 7a,b). The percentage of each cell category in each sample was demonstrated (Figure 7c). We found that the proportion of epithelial cells was Top 1 in each sample and was significantly different between CRC and control groups (Figure 7c). Subsequently, further investigation was conducted to assess the expression of prognostic genes in each cell. In the single-cell data profile, POU4F1, AIF1L, and SLC18A1 expression was detected, whereas HOXC6 and INSL4 were not detected in this dataset. Between CRC and control groups, the expression of AIF1L and SLC18A1 in epithelial cells was most significantly different (Figure 7d). CRC, a malignant neoplasm of epithelial origin, is intricately linked to the formation and evolution of epithelial tissue. Based on the above results, epithelial cells as key cells were for further study.

### 3.9. CNV Alterations and Gene Expression in Epithelial Subpopulations

**Figure 8:**
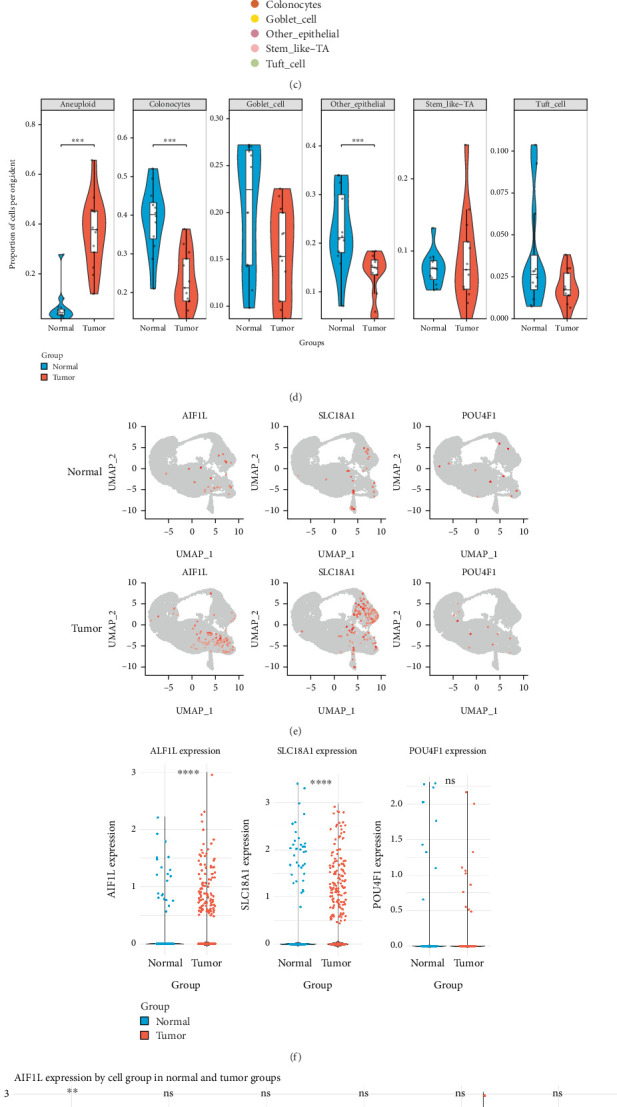
CNV inference, cell–cell communication, and gene expression analysis. (a) UMAP plot showing the clustering of epithelial cell subtypes. (b) Cell–cell communication network diagrams for normal (left) and tumor (right) groups. (c) UMAP plot showing CNV inference results based on epithelial cell subtypes. (d) Violin plots depicting the proportion of each epithelial cell subtype. (e) UMAP visualization of AIF1L, SLC18A1, and POU4F1 expression patterns. (f) Box plots comparing the expression levels of AIF1L, SLC18A1, and POU4F1. (g) Violin plots illustrating the expression of AIF1L and SLC18A1 in different epithelial subtypes. ns, not significant. ⁣^∗^*p* < 0.05, ⁣^∗∗^*p* < 0.01, ⁣^∗∗∗^*p* < 0.001, and ⁣^∗∗∗∗^*p* < 0.0001.

In the epithelial cell subpopulation analysis, UMAP clustering identified five distinct epithelial subtypes, including colonocytes, goblet cells, tuft cells, stem-like TA cells, and other epithelial cells (Figure 8a). Cell–cell communication analysis indicated that the number of interactions involving stem-like TA cells was higher in the tumor group than in the control group, with a notable increase in interactions between stem-like TA cells and fibroblasts (Figure 8b). CNV inference showed that aneuploid cells were mainly localized within the stem-like TA cell population (Figure 8c). The proportion analysis showed that colonocytes were reduced in tumor samples compared to normal tissues, whereas stem-like TA cells were increased (Figure 8d). The overall proportion of aneuploid cells in epithelial cell subpopulations was higher in tumor tissues than in normal tissues. Gene expression analysis revealed that AIF1L was upregulated in aneuploid cells, while SLC18A1 and POU4F1 showed no significant differences in expression within this population (Figures 8e, 8f, and 8g).

### 3.10. AIF1L May Promote Cell Proliferation and Migration in the MSI CRC

**Figure 9:**
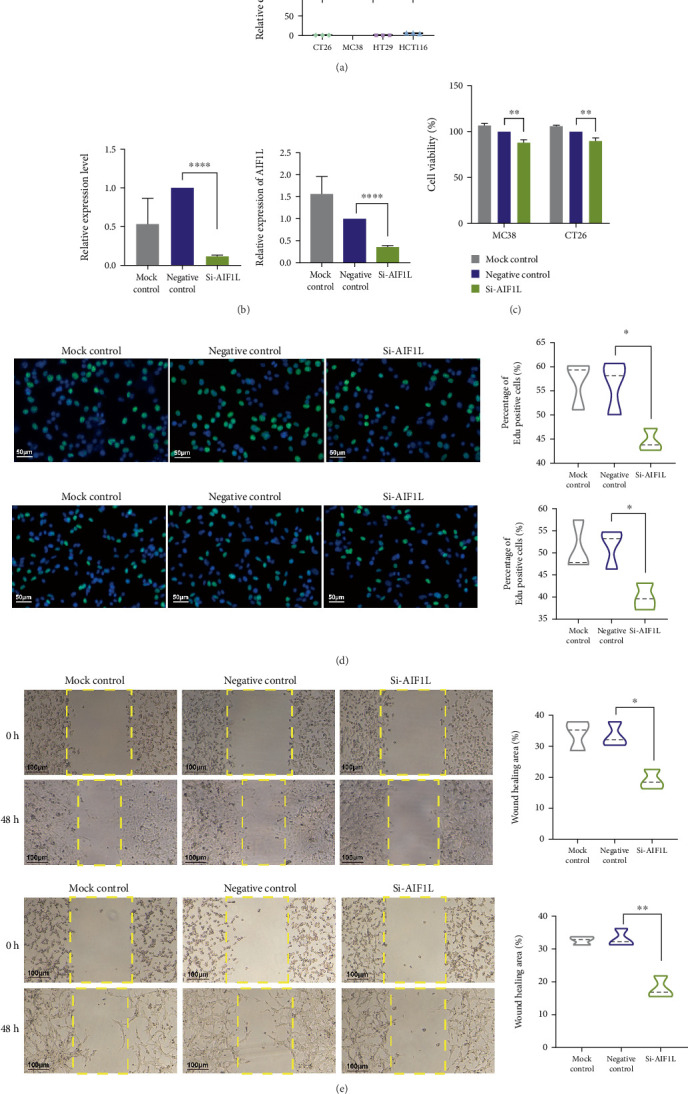
AIF1L enhances cell proliferation and migration in MSI CRC. (a) The expression of AIF1L in MSI-H (MC38 and HCT116) and MSS (CT26 and HT29) cell lines. (b) qRT-PCR to evaluate the level of AIF1L mRNA in MSI-H (MC38, left) and MSS (CT26, right) cell lines after transient transfection. (c) Assessment of cell proliferation by CCK8. (d) Assessment of cell proliferation by Edu. From top to bottom: MC38; CT26. (e) Assessment of cell migration. From top to bottom: MC38; CT26. ⁣^∗^*p* < 0.05, ⁣^∗∗^*p* < 0.01, ⁣^∗∗∗^*p* < 0.001, and ⁣^∗∗∗∗^*p* < 0.0001.

AIF1L was further examined for its biological role in MSI CRC through in vitro experiments. Initially, qRT-PCR was utilized to assess AIF1L expression in MSI-H and MSS cell lines, revealing that AIF1L expression levels were notably elevated in MSI-H cell lines compared to MSS cell lines (Figure 9a). We then assessed the expression level of AIF1L in the MSI-H (MC38) and MSS (CT26) cell lines after transient transfection by qRT-PCR and determined that siRNA interference across both cell lines led to significantly reduced expression levels of AIF1L (Figure 9b). To explore the impact of AIF1L on cell proliferation, CCK-8 and Edu were carried out. Results of both methods illustrated the reduced proliferative capacity of the cells in the Si-AIF1L group in both in MSI-H (MC38) and MSS (CT26) cell lines (Figure 9c,d). Finally, we also performed a scratch assay to measure the impact of AIF1L on cellular migratory capacity. Results revealed that scratch healing was markedly delayed in the Si-AIF1L group (Figure 9e). These results indicate that targeting AIF1L could be beneficial for MSI CRC treatment and may enhance patient prognosis.

### 3.11. AIF1L May Involve the Development of MSI CRC Through Ferroptosis

**Figure 10:**
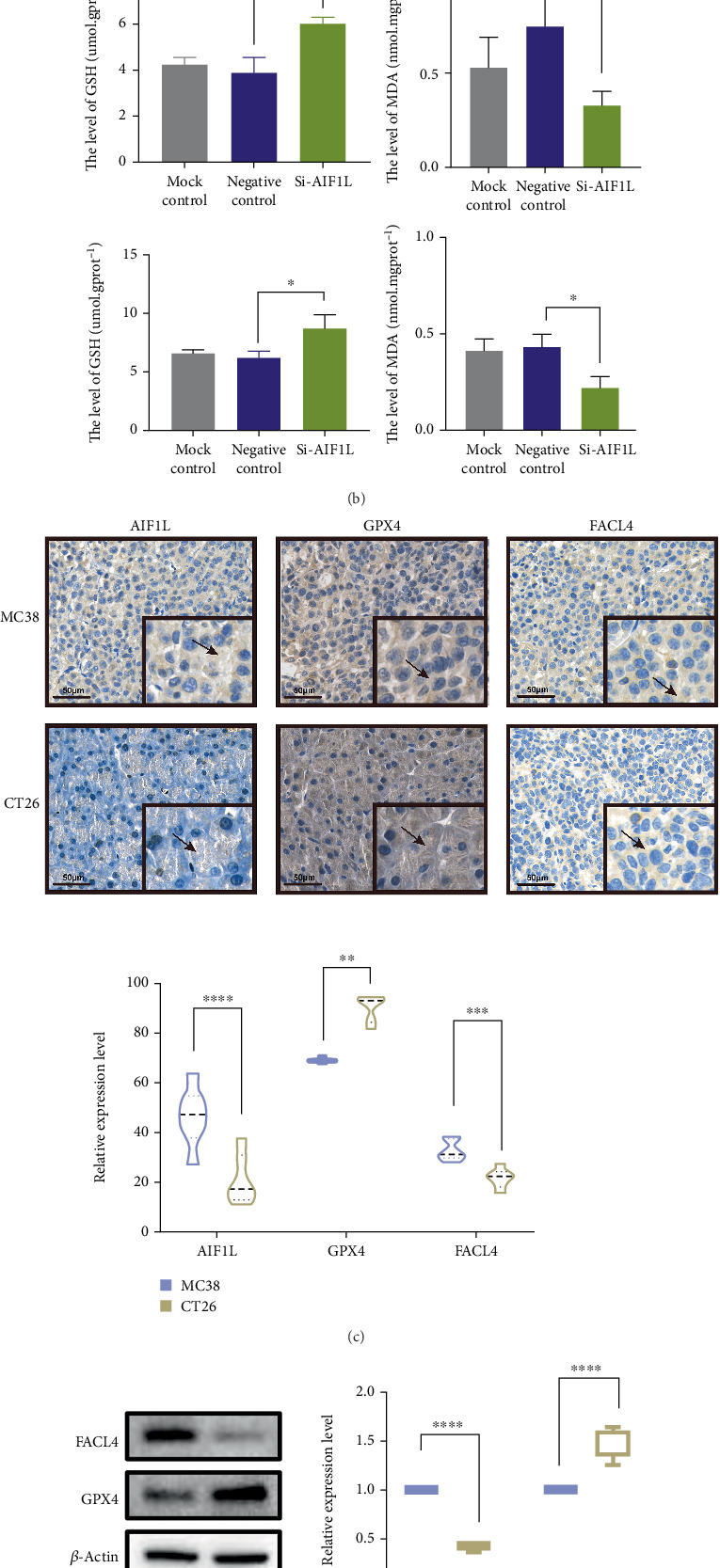
AIF1L may involve the development of MSI CRC through ferroptosis. (a) The expression levels of FACL4 and GPX4 in MSI-H and MSS cell lines. From top to bottom: MC38; CT26. (b) Assessments of MDA and GSH content in MSI-H and MSS cell lines. From top to bottom: MC38; CT26. (c) The protein expression for AIF1L, GPX4, and FACL4 in MSI-H and MSS tumor tissues through IHC analysis. (d) Western blot analysis of AIF1L and GPX4 protein levels in MSI-H and MSS mouse models. (e) Assessments of MDA and GSH content in MSI-H and MSS tumor tissues. ⁣^∗^*p* < 0.05, ⁣^∗∗^*p* < 0.01, ⁣^∗∗∗^*p* < 0.001, and ⁣^∗∗∗∗^*p* < 0.0001.

The above results revealed that AIF1L was highly expressed under MSI status and positively correlated with ferroptosis. To validate this, we initially assessed the expression of ferroptosis-related marker genes (FACL4 and GPX4) in the MSI-H (MC38) and MSS (CT26) cell lines. The results suggested that in both the MSI-H (MC38) and MSS (CT26) cell lines, FACL4 expression in the Si-AIF1L group was dramatically reduced, while GPX4 was substantially elevated compared to the negative control group (Figure 10a). We conducted assessments of MDA and GSH levels in the MSI-H (MC38) and MSS (CT26) cell lines, revealing that the MDA levels in the Si-AIF1L group were significantly lower than those in the negative control, although the GSH levels exhibited an opposite trend (Figure 10b). Additionally, we constructed the MSI-H and MSS mouse models. IHC experiments indicated that AIF1L and FACL4 exhibited higher expression in MSI-H mouse tumor tissue, whereas GPX4 demonstrated increased expression in MSS mouse tumor tissue (Figure 10c). The results of WB indicated that GPX4 expression was reduced, while FACL4 expression was elevated in MSI-H mouse tumor tissue (Figure 10d). Moreover, MSI-H mice exhibited elevated MDA levels and reduced GSH levels compared to MSS mice (Figure 10e). These findings indicate that AIF1L may promote the progression of MSI CRC by enhancing ferroptosis.

## 4. Discussion

Programmed cell death (PCD) plays a critical role in CRC progression and treatment response [24]. However, PCD in CRC involves a dynamic network of molecular interactions, and no single pathway fully explains its complexity [25]. To better understand the role of PCD in MSI CRC, we integrated gene sets from multiple PCD types and constructed a five-gene prognostic model comprising POU4F1, AIF1L, HOXC6, SLC18A1, and INSL4. These genes have been implicated in diverse cancer-related processes. POU4F1 was linked to tumor proliferation and migration [26], and AIF1L was associated with CRC risk modulation [27]. SLC18A1 may contribute to hepatic metastasis and CRC progression [28, 29]. INSL4 was part of an immunogenic cell death signature predicting favorable prognosis [30], and HOXC6 has been implicated in immune evasion and CD8^+^ T cell dysfunction through cytokine and checkpoint pathways [31, 32]. This model successfully stratified MSI CRC patients into distinct risk groups, with the high-risk group exhibiting significantly poorer prognosis, underscoring its potential clinical value as a prognostic tool. Furthermore, we integrated clinicopathological features (age, stage, and risk score) into a nomogram for predicting individual survival to enhance its clinical applicability. The calibration curves demonstrated good agreement with actual outcomes, and the DCA indicated a greater net benefit than using clinical features or risk score alone.

Among the five prognostic genes, AIF1L was prioritized for experimental validation based on its strong association with ferroptosis and its specific expression in relevant intestinal epithelial cells. Although both AIF1L and HOXC6 exhibited positive correlations with ferroptosis scores, single-cell data showed that HOXC6 was not expressed in intestinal epithelial cells, making AIF1L a more biologically relevant and experimentally tractable candidate. AIF1L expression was positively associated with ferroptosis driver genes and negatively correlated with suppressor genes. To validate its functional role, we performed siRNA-mediated knockdown of AIF1L in MSI-H (MC38) and MSS (CT26) CRC cell lines. Silencing AIF1L reduced ACSL4 expression and the lipid peroxidation marker MDA while increasing the antioxidant factors GPX4 and GSH. These results align with known mechanisms of ferroptosis resistance in CRC [33]. GPX4 and GSH are key antioxidant systems that suppress ferroptosis by reducing lipid peroxides, whereas MDA is a marker of lipid peroxidation and typically decreases when ferroptosis is suppressed [34]. In contrast, ACSL4 facilitates lipid peroxidation and promotes ferroptosis [35]. These changes indicate that AIF1L downregulation inhibits ferroptosis through modulation of lipid peroxidation and antioxidant capacity. Similar trends in tumor tissues further support its role as a ferroptosis regulator in MSI CRC. Beyond its association with ferroptosis, single-cell analysis highlighted that stem-like TA cells in tumor samples exhibited enhanced intercellular communication, particularly with fibroblasts. This interaction pattern may link epithelial–fibroblast crosstalk to extracellular matrix remodeling in cancer [36]. CNV inference further revealed enrichment of aneuploid epithelial cells within this subtype, suggesting a potential association between genomic instability and altered cell–cell communication in the tumor microenvironment.

Immune checkpoint therapy gained approval for treating patients with CRC exhibiting high MSI or mismatch repair defects [37]. Our study found that immune checkpoint gene expression was significantly higher in the MSI-H group than in the MSS group. One possible mechanism is that MSI-H tumors generally have a higher mutational burden, leading to the generation of more neoantigens, which in turn activates the immune system and induces the upregulation of immune checkpoint molecules [38]. Notably, although the high-risk group identified by our model largely overlapped with MSI-H patients who are traditionally responsive to ICIs, the TIDE algorithm predicted a stronger immunotherapy response in the low-risk group. This apparent discrepancy may stem from the predictive limitations of TIDE in the context of MSI CRC. Since the TIDE model was primarily trained on pancancer cohorts, it may not fully capture the unique immune microenvironmental features of MSI CRC, thereby affecting its applicability to this specific population [39]. Therefore, caution is warranted when interpreting TIDE results in the context of MSI status. Meanwhile, cancer can establish an immunosuppressive environment. It impairs the immune response and leads to immunotherapy resistance [40]. Nonetheless, the immune system could mitigate this by triggering ferroptosis in neoplastic cells, a mechanism mostly facilitated by CD8^+^ T lymphocytes. These cells promote lipid peroxidation via the interferon-*γ* (IFN-*γ*) pathway and activate FACL4, thereby altering tumor lipid composition and enhancing the efficacy of ICIs [41]. In our study, AIF1L was closely associated with activated CD8 T cells and involved in immune-related pathways, such as “chemokine signaling pathway,” “T cell receptor signaling pathway,” and “Toll-like receptor signaling pathway.” These suggest that AIF1L may enhance the cytotoxic function in MSI CRC. Additionally, AIF1L was significantly linked to monocytes and Tfh cells. Monocytes play a pivotal role in antigen presentation and immune activation [42], while Tfh cells are essential for B cell-mediated immunity [43]. Meanwhile, the correlation between AIF1L and IL10RA indicated its potential role in modulating inflammatory responses [44]. It is possible that AIF1L indirectly regulates tumor immune responses by altering ferroptosis susceptibility in immune cells. These findings highlight AIF1L as a crucial link between ferroptosis and immune regulation, providing insights into its potential as both a biomarker and therapeutic target in MSI CRC.

While AIF1L appears to enhance ferroptosis susceptibility and activate immune function in CRC, its role in tumor progression seems to be context-dependent. Previous studies have shown that the expression level of AIF1L in thyroid cancer tissues is significantly lower than that in normal tissues, and it has the ability to inhibit the migration and invasion of thyroid cancer cells [45]. A similar effect has been demonstrated in breast cancer. The absence of AIF1L contributes to increased cell migration, invasion, and poor prognosis, highlighting its role in regulating cancer cell mobility [46]. Our study also shows that AIF1L may promote cell migration in the MSI CRC. Although the specific mechanism of AIF1L in promoting tumor metastasis and invasion is not fully understood, existing studies have found that AIF1L can regulate cell motor capacity. It is reported that in human podocyte lines, the loss of AIF1L may lead to increased filopodia formation and decreased actomyosin contractility, which may provide more opportunities for tumor cells to migrate [47]. Nevertheless, further studies are still needed to evaluate whether targeting AIF1L could provide therapeutic benefits by balancing its ferroptosis-promoting effects while mitigating potential prometastatic activity.

This study has certain limitations. Our study relies on publicly available datasets, which may introduce potential publication bias. In addition, the current study also lacks in vivo validation of AIF1L knockdown, and further investigations are needed to clarify its role in ferroptosis. Recent work has explored the use of multimodal learning and large language model chatbots in digestive endoscopy [48], highlighting the growing role of artificial intelligence in gastroenterology research. Finally, while our approach demonstrates potential, additional confirmation of its efficacy in prospective clinical studies is required.

## 5. Conclusion

In summary, we conducted and validated the cell death signature risk model based on TCGA and GEO databases. Five prognostic genes (POU4F1, AIF1L, SLC18A1, INSL4, and HOXC6) for MSI CRC patients were identified, and AIF1L may contribute to the development of MSI CRC by promoting ferroptosis, which may facilitate clinical prognostic evaluation and personalized management in MSI CRC.

## Data Availability

The datasets analyzed during the current study are publicly available as follows: TCGA-COAD and TCGA-READ transcriptomic and clinical data were retrieved from TCGA (https://portal.gdc.cancer.gov/repository). GEO dataset was retrieved from GSE29621 (https://www.ncbi.nlm.nih.gov/geo/query/acc.cgi?acc=GSE29621) and GSE166555 (https://www.ncbi.nlm.nih.gov/geo/query/acc.cgi?acc=GSE166555). Further processed data or analysis scripts are available from the corresponding authors upon reasonable request.

## Supporting Information

Supporting Information 1 Figure S1: Construction of animal model.

Supporting Information 2 Figure S2: GSEA of SLC18A1, POU4F1, and INSL4. GO enrichment analysis of (A) SLC18A1, (B) POU4F1, and (C) INSL4. KEGG enrichment analysis of (D) SLC18A1, (E) POU4F1, and (F) INSL4.

Supporting Information 3 Table S1: The primer sequences of AIF1L in different species.

Supporting Information 4 Table S2: List of GO items of DEGs enriched.

Supporting Information 5 Table S3: List of KEGG pathways of DEGs enriched.

Supporting Information 6 Table S4: List of AIF1L-related immune cells and marker genes.

Supporting Information 7 Table S5: List of ferroptosis marker genes.

Supporting Information 8 Table S6: List of ferroptosis driver genes.

Supporting Information 9 Table S7: List of ferroptosis suppressor genes.
