# Supplementary figures and images for: AIF1L as a Ferroptosis-Linked Biomarker in Microsatellite States–Driven Colorectal Cancer: Functional and Diagnostic Insights From Multiomics Analysis
Source: Hum Mutat. 2025 Oct 10;2025:6663166. doi: 10.1155/humu/6663166 (PMC12534154; doi:10.1155/humu/6663166)

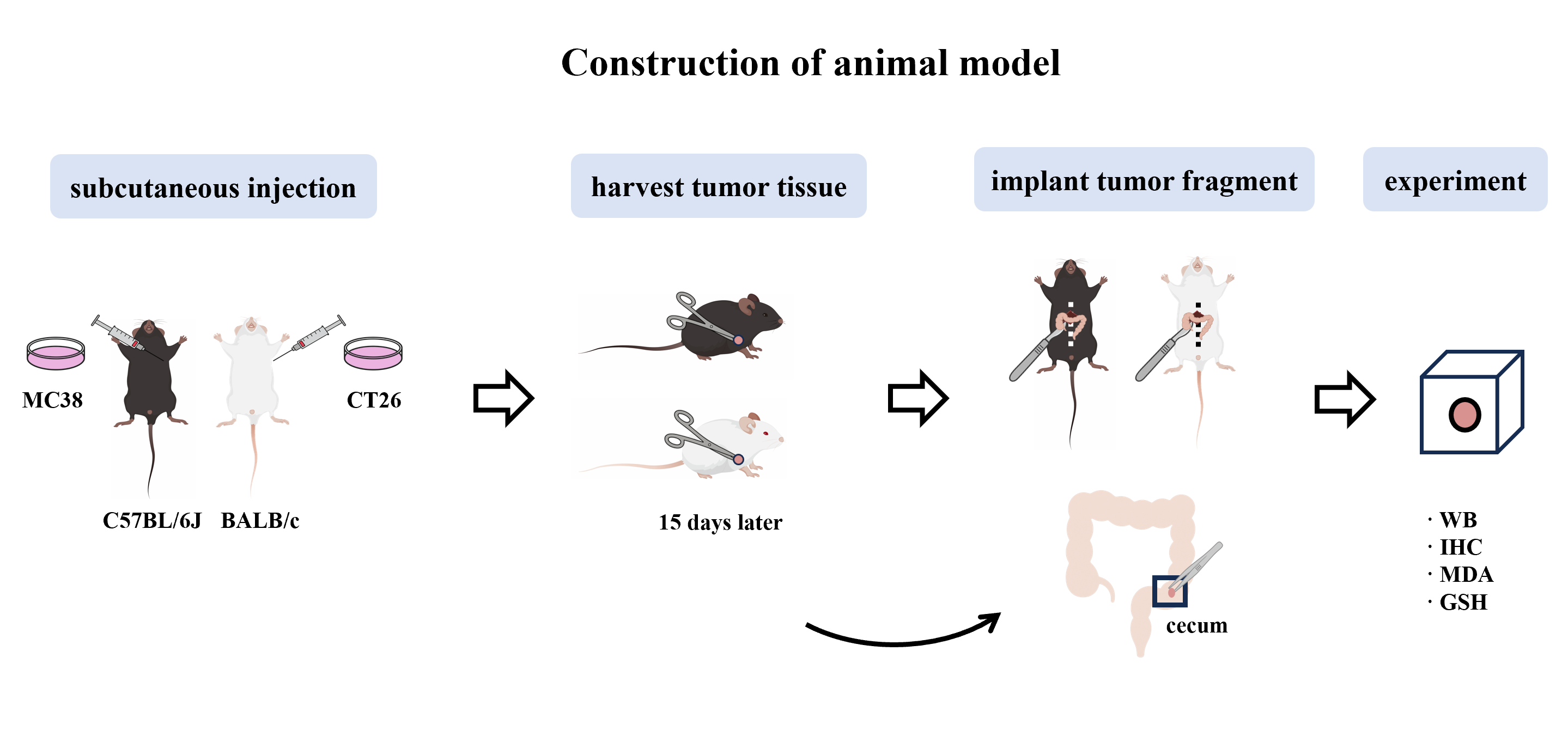

Supplement: Supporting Information 1 — Figure S1: Construction of animal model. [file 6663166.f1.tif]
