# Supplementary material for: AIF1L as a Ferroptosis-Linked Biomarker in Microsatellite States–Driven Colorectal Cancer: Functional and Diagnostic Insights From Multiomics Analysis
Source: Hum Mutat. 2025 Oct 10;2025:6663166. doi: 10.1155/humu/6663166 (PMC12534154; doi:10.1155/humu/6663166)
Supplement: Supporting Information 3 — Table S1: The primer sequences of AIF1L in different species. [file 6663166.f3.docx]

Supplementary Table 1 The primer sequences of AIF1L in different species

| Gene | Primer sequence （5’-3’） | Species |
| --- | --- | --- |
| AIF1L | F:CAAGACCCACCTGGAGATGAAGAAG | Homo sapiens |
|  | R:CTTGAGGACAGCCGACCGTTTC |  |
| β-Actin | F:CTTCCAGCCTTCCTTCCTGG | Homo sapiens |
|  | R:CTGTGTTGGCGTACAGGTCT |  |
| AIF1L | F:AGCGGTCGGCTGTCCTCAAG | Mus musculus |
|  | R:CAAGGCAGGCTGGCGATGTC |  |
| β-Actin | F: ACTGCCGCATCCTCTTCCTC | Mus musculus |
|  | R: AACCGCTCGTTGCCAATAGTG |  |
